# Viral tropism for the testis and sexual transmission

**DOI:** 10.3389/fimmu.2022.1040172

**Published:** 2022-11-09

**Authors:** Fei Wang, Jing Zhang, Yu Wang, Yongmei Chen, Daishu Han

**Affiliations:** Institute of Basic Medical Sciences, Chinese Academy of Medical Sciences, School of Basic Medicine, Peking Union Medical College, Beijing, China

**Keywords:** virus, testis, immune privilege, innate antiviral response, sexual transmission

## Abstract

The mammalian testis adopts an immune privileged environment to protect male germ cells from adverse autoimmune reaction. The testicular immune privileged status can be also hijacked by various microbial pathogens as a sanctuary to escape systemic immune surveillance. In particular, several viruses have a tropism for the testis. To overcome the immune privileged status and mount an effective local defense against invading viruses, testicular cells are well equipped with innate antiviral machinery. However, several viruses may persist an elongated duration in the testis and disrupt the local immune homeostasis, thereby impairing testicular functions and male fertility. Moreover, the viruses in the testis, as well as other organs of the male reproductive system, can shed to the semen, thus allowing sexual transmission to partners. Viral infection in the testis, which can impair male fertility and lead to sexual transmission, is a serious concern in research on known and on new emerging viruses. To provide references for our scientific peers, this article reviews research achievements and suggests future research focuses in the field.

## 1 Introduction

The male reproductive system (MRS) is composed of various organs with relatively separate anatomical positions, highly organized microenvironments, multifarious cell types and distinct molecular milieu for the execution of different functions. Sperm is produced in the testis and stored in the epididymis. Before ejaculation, sperm must joint with the seminal plasma (SP) mainly secreted by the seminal vesicle and prostate. Notably, sperm are immunogenic because they are initially produced after the establishment of central immune tolerance. To protect sperm from an adverse immune response, the testis and epididymis adopt immune privileged microenvironments (1, 2). However, the immune privileged environment also provides sanctuaries for microbial pathogens escaping from immune surveillance. The MRS can frequently be infected by a broad spectrum of microorganisms, including viruses, bacteria, and parasites (3). To overcome this immune privileged status and mount an efficient local defense against invading microbes, tissue-specific cells are well equipped with pattern recognition receptors (PRRs). PRR-initiated innate immune responses within local cells play important roles in the MRS defense against microbial infections (4). However, immune homeostasis in the MRS can be disrupted by microbial infection and autoantigens, resulting in inflammatory conditions in the MRS, which are important etiological factors of male subfertility and infertility (5).

In particular, the immune privileged environment in the testis can be hijacked by a large number of viruses (6). Moreover, the viruses that infect the MRS may shed to semen, thereby leading to sexual transmission. Emerging epidemic viruses in recent years have raises important concerns regarding viral reservoirs in the MRS and their impacts on male fertility, as well as the possibility of sexual transmission. However, only a minority of viruses that infect the testis seriously impact male fertility, which can be due to the restriction of viral replication by testicular cells with innate antiviral machineries (7). While more than thirty virus types have been detected in the semen, only half of them are sexually transmitted (6). Moreover, the efficiency of viral sexual transmission is generally low, even for the typical sexually transmitted viruses (8). In addition to an association between sexual transmission and viral load in the semen and mucosal barrier of the anogenital tract (9), growing evidence indicates that SP impacts viral infection (10). This review article will discuss testicular immune environment, viral effect on male fertility and sexual transmission of viruses.

## 2 Immune privilege of the testis

Immune privilege is defined as a special immune microenvironment where the allo- and auto-antigens are tolerated (11). The pregnant uterus, eye, brain and testis are typical immune privileged organs in human beings (12). An immune privileged status is essential for the protection of auto-antigens from adverse immune responses.

**Figure 1:**
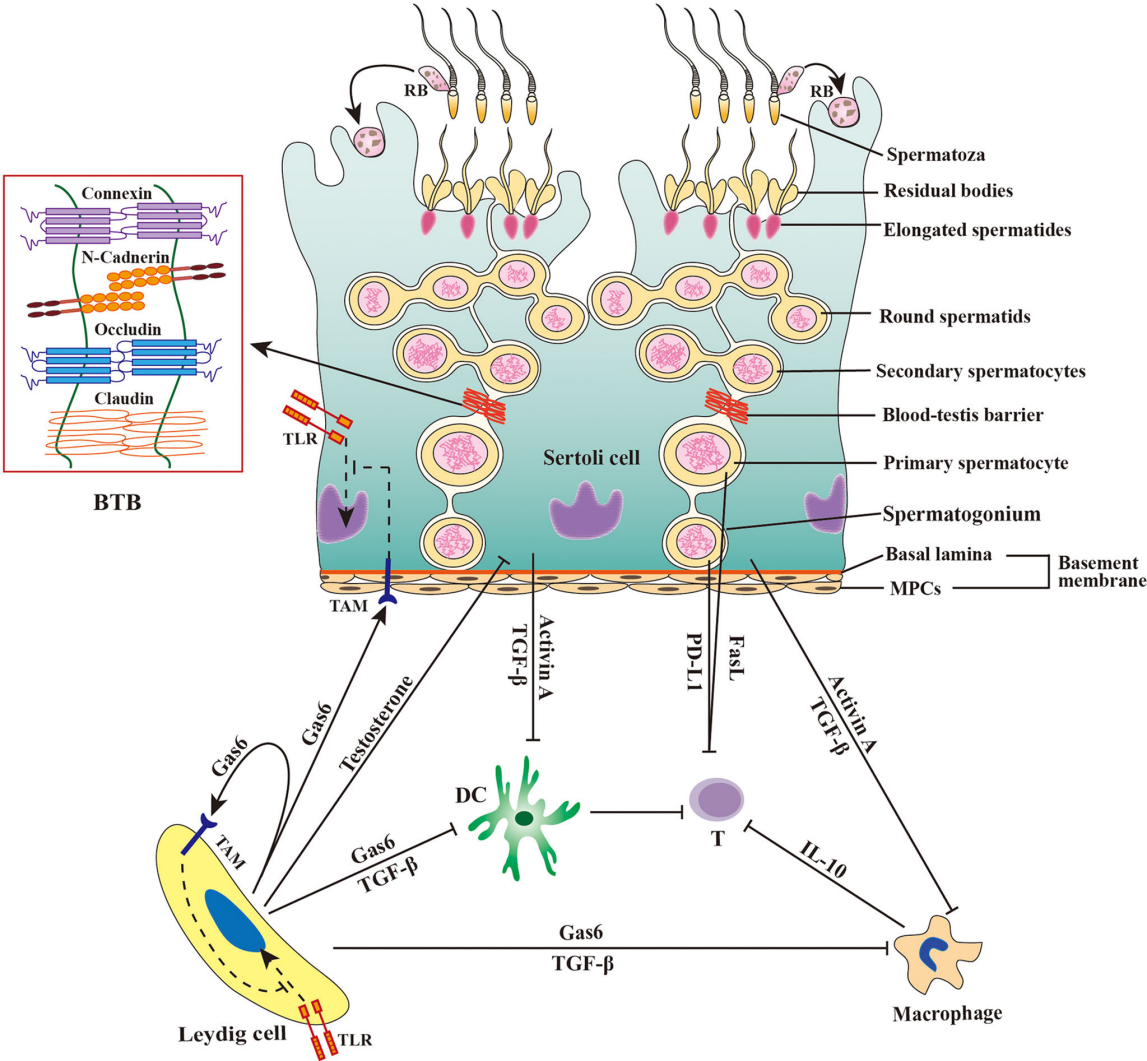
Schematic of immune privilege in the testis. The immune privilege of the testis is regulated by the tissue structure and anti-inflammatory factors. The blood-testis barrier (BTB) separates the late stage of germ cells from immune cells in the interstitial spaces. Sertoli cells secrete activin A and transforming growth factor β (TGF-β) that inhibit the immune responses of dendritic cells (DCs) and macrophages. Leydig cells produce growth-arrest-specific gene 6 (Gas6), TGF-β, and testosterone, which inhibit the immune responses of DCs, macrophages, and Sertoli cells. Male germ cells produce Fas ligand (FasL) and programmed death-ligand 1 (PD-L1), and both FasL and PD-L1 induce apoptosis of T lymphocytes (T). Macrophages exhibit immunosuppressive properties by producing anti-inflammatory cytokine IL-10 to inhibit effective T lymphocyte responses. Sertoli cells and Leydig cells express Tyro3, Axl and Mer (TAM) receptor tyrosine kinases, which can be activated by Gas6 and inhibit toll-like receptor (TLR)-initiated innate immune responses. MPCs, myoid peritubular cells; RB, residual body; ⟂, inhibition.

The mammalian testis produces a large number of sperm after puberty when the central immune tolerance has already been established during fetal and neonatal periods. Therefore, sperm are immunogenic and induce immune responses (13). In fact, a majority of male germ cells prior to developing to sperm are also immunogenic (14). The immune privilege of the testis, which is maintained by the orchestration of tissue structure, cellular composition and molecular milieu (**Figure 1**) (1), protects male germ cells and allografts from immune rejection (15, 16).

### 2.1 Histological structure for immune privilege of the testis

The mammalian testis is relatively isolated from the main body and has a lower temperature than the rest of the body. In histology, the testis is highly compartmentalized into two regions, the seminiferous tubule and interstitial spaces, which are separated by the tubular basement membrane composed of the basal lamina and myoid peritubular cells (MPCs) enclosing the seminiferous tubules. Spermatogenesis takes place within the seminiferous tubule, which is divided into basal and adluminal compartments by the blood-testis barrier (BTB) formed between adjacent Sertoli cells (SCs) near the basement membrane. Testosterone is synthesized by Leydig cells (LCs) in the interstitial spaces. The BTB is believed to be responsible for testicular immune privilege because it can efficiently sequester immunogenic substances produced by late-stage testicular germ cells (TGCs) in the adluminal compartment and immune component in the interstitial spaces. This concept has been challenged by the observation that the early-stage germ cells, which are localized outside the BTB, also produce immunogenic antigens (16). The basement membranes may also restrict the entry of immune cells into the seminiferous tubules, since immune cells are not found in both the basal compartment. The potential role of the basement membranes in the immune privilege within the seminiferous tubules is yet to be confirmed. Moreover, the interstitial spaces also enjoy the immune privilege because xenografts in this region are protected from immune rejection (17). Therefore, the BTB and basement membranes would not be fully responsible for the testicular immune privilege. Substantial evidence supports that multifarious testicular cells and immunosuppressive factors produced by testicular cells also play crucial roles in maintaining immune privileged status.

### 2.2 Cellular and molecular involvements in the testicular immune privilege

The main function of the testis is spermatogenesis, a developmental process of TGCs from spermatogonia to spermatozoa. Therefore, different developing stages of TGCs represent the majority of testicular cells in adults. Since the first round of spermatogenesis is completed after puberty when immune tolerance has been already established, most TGCs express immunogenic antigens. Testicular immune privilege is essential to protect germ cells from immune responses. Notably, TGCs express Fas ligand (FasL) (18). Since FasL triggers a key immune tolerance signaling pathway (19), TGC-expressed FasL potentially plays a role in maintaining testicular immune privileged status, which is worthy of clarification.

Besides TGCs, SCs are the only somatic cells within the seminiferous tubules. SCs form the BTB using the various cell junctions between two adjacent SCs (7). SCs tightly embrace developing TGCs to create a microenvironment necessary for spermatogenesis. SCs also play important roles in regulating testicular immune privileged status through multiple mechanisms (20, 21). SCs can protect xenografts from immune rejection without the formation of a physical barrier after co-transplantation, suggesting that these cells inhibit immune responses independent of the BTB (22, 23). During TGC development, most TGCs undergo apoptosis and healthy germ cells shed their major cytoplasmic portions as residual bodies before the formation of complete sperm. The apoptotic germ cells and residual bodies must be removed in a timely manner by SCs through phagocytosis, which prevent immunogenic antigens to be released from the breakdown of apoptotic cells and residual bodies (24). Furthermore, SCs secrete multiple immunosuppressive factors, such as activin A and transforming growth factor β (TGF-β), that play roles in maintaining the immune privileged status (25, 26).

MPCs build the outer layer of the basement membrane and perform contractile activity to promote the transportation of spermatozoa into the epididymis (27). Human MPCs produce various immunoregulatory cytokines, including TGF-β, monocyte chemotactic protein 1 (MCP-1) and leukemia inhibitory factor, which regulate immune responses in the testis (28). MPCs also express tumor necrosis factor-α (TNF-α) receptor to mediate IL-6 production (29). These observations suggest that MPCs should regulate the testicular immune environment in a paracrine manner. The potential roles of MPCs in regulating testicular immunity have been rarely explored.

LCs belong to a major cell population in the interstitial spaces and synthesize more than 90% testosterones in men. LCs also regulate the testicular immune environment using different mechanisms (30). LCs restrict lymphocyte and macrophage numbers in the testis (31, 32). Testosterone produced by LCs is essential for maintaining the testicular immune privileged status, which (33, 34). Recent studies have demonstrated that LCs produce growth arrest-specific gene 6 (Gas6), which inhibits innate immune responses in testicular somatic cells through negatively regulating toll-like receptor (TLR) signaling (35).

Testicular macrophages (TMs) represent the major leukocytes in the testicular interstitial spaces. There are two types of TMs. The majority of TMs belong to M2-like testicular resident macrophages that express CD163 and exhibit immune inhibitory activities (36). In addition, minor M1-like macrophages that highly express CD63 can be found in the testis, which are newly migrated from peripheral circulation and display pro-inflammatory phenotypes. In the rat orchitis model, the ratio of these two types of macrophages can be remarkably changed (37). TMs predominantly produce high level of interleukin 10 (IL-10) and low levels of TNF-α upon the activation by antigens (38, 39). TMs inhibit T-cell activation and induce the differentiation of naïve T-cells into immunosuppressive regulatory T cells (Tregs) (39, 40). TMs closely interact with LCs and regulate LC development and function (41). TMs predominantly reduce immune response commitment to the testicular immune privileged environment (42).

In addition to macrophages, other minor leukocytes, including dendritic cells, lymphocytes, and mast cells, can be also found in the interstitial spaces under physiological conditions. Dendritic cells (DCs) are antigen-presenting cells that induce lymphocyte activation and differentiation. DCs activate lymphocyte immune responses to foreign antigens while suppressing T-cell responses to self-antigens. DCs account for a small proportion of rat testicular interstitial cells under physiological conditions, and can be markedly increased during EAO induction (43). DCs in normal rat testis are unable to stimulate naïve T-cell proliferation (43, 44), suggesting that DCs favor the immune privilege status of the testis. Lymphocytes account for about 15% of the adult rat testicular immune cells, and the lymphocytes were mainly CD8^+^ cells and a small number of CD4^+^ cells (32). In the EAO model and in autoimmune infertility patients, testicular lymphocytes were dramatically increased (45). Recent studies showed that Tregs efficiently suppress immune responses in the testis and regulates efficiently suppress immune responses in the testis and regulate immune tolerance to sperm antigens (46). Leukocyte numbers are significantly increased under inflammatory conditions, suggesting these cells are involved in inflammatory pathogenesis (47). Taken together, the testicular immune privileged status is maintained by multiple mechanisms at the tissue structural, cellular and molecular levels (**Figure 1**).

## 3 Viral tropism for the testis

While the immune privileged status is essential for the execution of testicular functions, it also provides a sanctuary for microbial pathogens to escape immune surveillance. In particular, a large spectrum of viruses have a tropism for the testis. The testis may serve as a viral reservoir and impair testicular functions.

**Figure 2:**
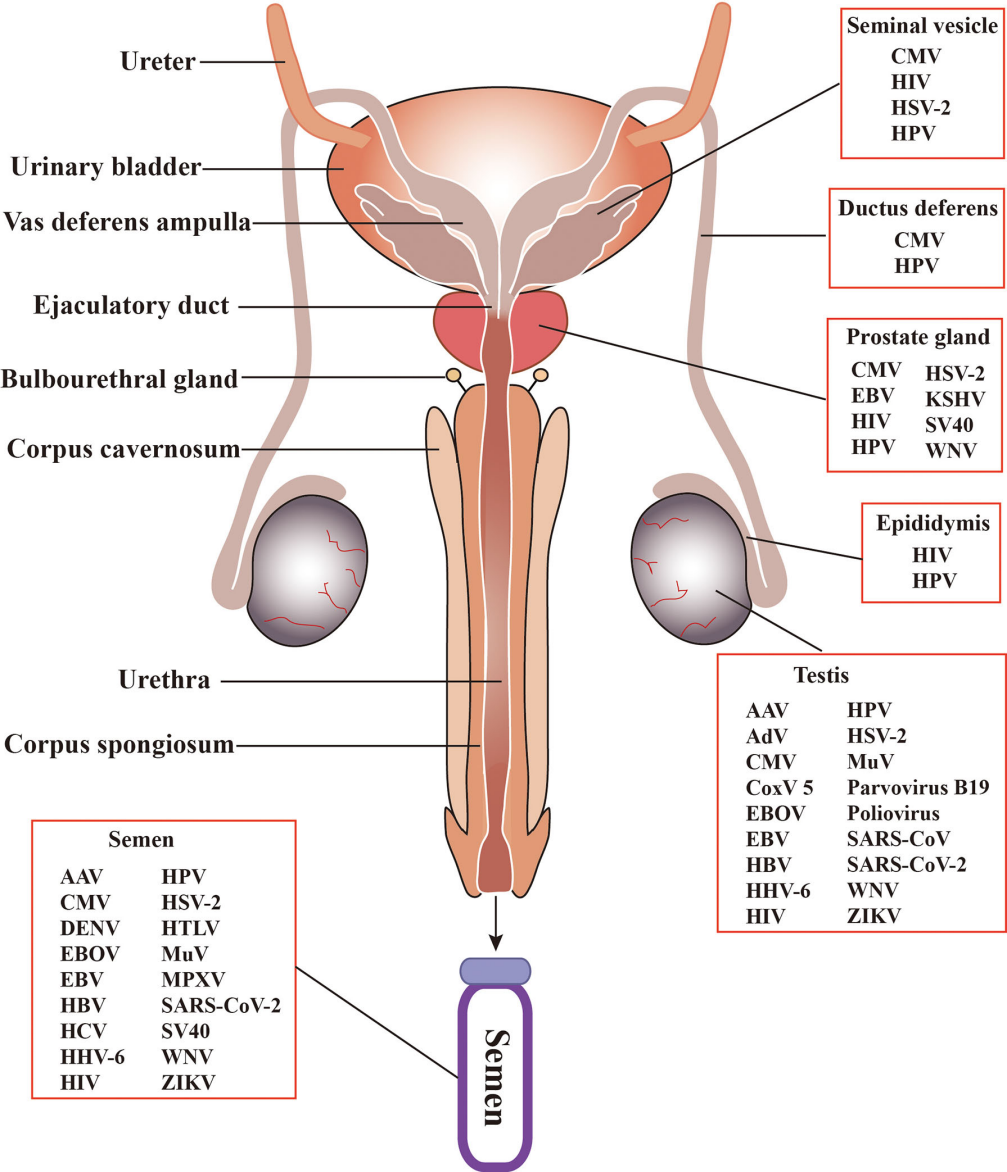
Schematic of the male reproductive system (MRS) and virus distribution. The MRS is composed of various organs, including testes, epididymides, the prostates, seminal vesicles, and bulbourethral gland. These organs are connected by genital ducts, including ductus deferens, vas deferens ampulla, ejaculatory duct and urethra. A broad spectrum of viruses have been detected in the MRS and in semen. AAV, Adeno-associated virus; AdV, Adenovirus; CMV, Cytomegalovirus; CoxV 5, Coxsackie virus 5; DENV, Dengue virus; EBV, Epstein-Barr virus; EBOV, Ebola virus; HBV, Hepatitis B virus; HCV, Hepatitis C virus; HHV-6, Human herpes virus -6; HIV, human immunodeficiency virus; HPV, human papilloma virus; HSV, Herpes simplex virus; HTLV, Human T lymphotropic virus; KSHV, Kaposi sarcoma associated herpes virus; MuV, mumps virus; MPXV, Monkeypox virus. SARS-CoV, severe acute respiratory syndrome associated-coronavirus; SV40, Simian virus 40; WNV, West-Nile virus; ZIKV, Zika virus.

The MRS is composed of various organs, including the testis, epididymis, seminal vesicle, prostate, bulbourethral gland, and penis (**Figure 2**). These organs are connected by several ducts, including the efferent ducts between the testis and epididymis, vas deferens between the epididymis and seminal vesicle, and urinary duct among the seminal vesicle, prostate, bulbourethral gland, and penis. The whole MRS can be infected by viruses (**Figure 2**). In particular, more than two dozens virus types have been detected in the human or animal testis (**Table 1**), suggesting that the testis is a major target of viruses in the MRS. However, only those viruses that may seriously impair male fertility or that can be sexually transmitted have been investigated for their host cells, impact on male fertility, and sexual transmission. Notably, recent emerging viruses raise great concern regarding their infection of the MRS and sexual transmission.

**Table 1:** Major viruses in the male reproductive system.

Viruses	Targets			Pathogenesis	Shedding in semen	Sexual transmission
	Human	NHP	Rodents			
**AAV**	Testis	/	/	Subfertility	Y	Y
**AdV**	Testis	/	/	Unclear	N	N
**CMV**	Testis, Vas deferens, Seminal vesicle, Prostate	/	/	Infertility	Y	Y
**CoxV B5**	Testis	/	/	Orchitis	N	N
**DENV**	/	/	/	Reversible alterations in sperm	Y	Y
**EBOV**	Testis	Seminal vesicle, Prostate	/	Testis as an anatomic reservoir for persistence	Y	Y
**EBV**	Testis, Prostate	/	/	Unclear	Y	Y
**HBV**	Testis, Penis	/	/	Sperm parameter alteration and infertility	Y	Y
**HCV**	/	/	/	Sperm parameter alteration and infertility	Y	Y
**HEV**	/	Testis, Epididymis	Testis	Testicular dysfunction	N	N
**HHV-6**	Testis	/	/	Unclear	Y	Y
**HIV**	Testis, Epididymis, Seminal vesicle, Prostate, Penis	/	/	Orchitis, “Sertoli-cell only” syndrome, and infertility	Y	Y
**HPV**	Testis, Epididymis, Vas deferens, Seminal vesicle, Prostate, Penis	/	/	Subfertility and infertility	Y	Y
**HSV**	Human: Testis, Prostate, Penis	/	Testis, Epididymis, Vas deferens, Seminal vesicle	Prostatitis, epididymitis, infertility, and sperm parameter alteration	Y	Y
**HTLV**	NA	/	/	Unclear	Y	Y
**Influenza virus**	NA	/	/	Sperm parameter alteration	N	N
**LCMV**	Testis	/	/	Unclear	N	N
**MuV**	Testis	/	/	Epididymo-orchitis and infertility	Y	N
**MPXV**	NA	/	/	Unclear	Y	Y
**Parvovirus**	Testis	/	/	Testicular germ cell tumors	N	N
**B19**
**Poliovirus**	Testis	/	/	Unclear	N	N
**SARS-CoV**	Testis	/	/	Orchitis	N	NA
**SARS-CoV-2**	Testis	/	/	Orchitis	Y	NA
**SV40**	Prostate	/	/	Unclear	Y	N
**WNV**	Testis, Prostate	/	/	Orchitis	Y	Y
**ZIKV**	Testis,	/	Epididymis, Vas deferens, Seminal vesicle, Prostate	Orchitis, epididymo-orchitis,	Y	Y
				and infertility in mouse		
				models. Sperm parameter		
				alteration in men		

AAV, Adeno-associated virus; AdV, Adenovirus; CMV, Cytomegalovirus; CoxV B5, Coxsackie virus B5; DENV, Dengue virus; EBV, Epstein-Barr virus; EBOV, Ebola virus; HBV, Hepatitis B virus; HCV, Hepatitis C virus; HEV, Hepatitis E virus; HHV-6, Human herpes virus -6; HIV, human immunodeficiency virus; HPV, human papillomavirus; HSV, Herpes simplex virus; HTLV, Human T-lymphotropic virus; LCMV, lymphocytic choriomeningitis virus; MuV, mumps virus; MPXV, Monkeypox virus. SARS-CoV, severe acute respiratory syndrome-associated-coronavirus; SV40, Simian virus 40; WNV, West-Nile virus; ZIKV, Zika virus. Y, Yes; N, No; NA, not available. NHP, non-human primate.

### 3.1 Human immunodeficiency virus (HIV)

HIV-1 has been intensively studied in the testis due to its typical sexual transmission and adverse effect on testicular functions (48). The testis is an HIV-1 sanctuary (49). However, HIV-1 does not infect tissue-specific cells in the testis because its infection strictly depends on its receptor CD4 and co-receptors CCR5 and CXCR4, which are absent in the testicular cells. Primary HIV-1 target cells are CD4^+^ lymphocytes and certain populations of macrophages and dendritic cells. Accordingly, there is no evidence that HIV infects testicular somatic cells. It is controversial whether HIV-1 internalizes or binds to male germ cells and uses germ cells as reservoirs to escape immune surveillance and antiviral drugs. There is convincing evidence that HIV-1 can be transmitted *via* spermatozoa as passive vectors (50). Several early studies demonstrated that HIV-1 particles and viral DNA can be detected in TGCs, including spermatozoa, suggesting that HIV-1 is internalized and replicated within TGCs (51–53). Moreover, HIV-1 DNA and simian immunodeficiency virus (SIV) have been found in human spermatozoa and monkey spermatogonia, respectively, confirming that HIV-1 and SIV are internalized into TGCs (54, 55). It is intriguing, however, to speculate how HIV-1 and SIV gain entry into TGCs in the absence of their receptors. Some studies have shown that galactoglycerolipid and mannose receptors mediate HIV-1 infection of TGCs (56, 57). It is interesting to clarify whether distinct mechanisms underlying HIV-1 infection exist in leukocytes and TGCs. Unfortunately, these issues have yet to be intensively investigated partially due to the poor survivability of TGCs *in vitro*. HIV infection of male germ cells and the underlying mechanisms require further clarification, which requires improvements in the culture system to prolong the survival of germ cells *in vitro*.

### 3.2 Mumps virus (MuV)

MuV has a high tropism for the testis and typically induces orchitis in addition to an inflammation in the parotid salivary glands (58). While MuV-induced orchitis severely impairs testicular functions and is a major etiological factor of male infertility, only one case report about a half century ago has shown the presence of MuV in the testis of a patient (59). Since MuV infection is mostly self-limiting and does not cause death, it is difficult to obtain human samples of MuV orchitis to investigate mechanisms by which MuV induces orchitis. In fact, the pathogenesis of MuV infection is mostly studied using animal models (60). Various animal models have been developed to study the pathogenesis after MuV infection (61, 62). We recently investigated mechanisms underlying MuV infection of testicular cells and impairment of testicular function using a mouse model (63).

While the manifestations of MuV infection are human diseases, MuV also infects the majority of mouse testicular cells. Our recent study demonstrated that sialic acid on the surface of mouse SCs and LCs plays a critical role in mediating MuV internalization into cells, whereas Axl and Mer receptor tyrosine kinases facilitate MuV replication within these cells through the inhibition of cellular innate antiviral responses (64). In addition to SCs and LCs, MuV also infects murine TMs and TGCs. Testicular cells employ different innate antiviral responses for restricting MuV replication after MuV infection (65). MuV replicates relatively rapidly in SCs compared to LCs and TMs but does not replicate in TGCs. Accordingly, LCs express relatively high levels of type 1 interferons (IFN-α and IFN-β) and antiviral proteins compared to SCs in response to MuV infection. TMs limit MuV replication through the induction of IFNs, antiviral proteins, and autophagy. TGCs do not express IFNs and antiviral proteins, and these cells may defend against MuV using autophagy (65).

PRRs are a large family of receptors that recognize conserved molecular patterns of microbial pathogens and initiate innate immune responses, which comprise the body’s first line of defense against microbial infection (66). PRR-initiated innate immune responses may also result in harmful inflammatory conditions (67). To understand mechanisms by which MuV induces orchitis, PRR-initiated innate immune responses in major testicular cells after MuV infection have been studied (68). MuV induces the expression of proinflammatory cytokines, including TNF-α, IL-6, MCP-1, and CXCL10, through the activation of TLR2 in SCs and LCs. MuV also triggers retinoic acid-inducible gene I (RIG-I) signaling that induces the production of IFN-α and IFN-β in SCs and LCs. Notably, SCs express relatively high levels of proinflammatory cytokines compared to LCs after MuV infection. The proinflammatory cytokines may lead to inflammatory conditions by recruiting and activating leukocytes in the testis. Therefore, SCs should contribute to orchitis after MuV infection. In contrast, LCs express high levels of IFNs and antiviral proteins in response to MuV infection, thereby exhibiting a stronger antiviral ability than SCs (68). However, MuV orchitis is not induced in mice, which may be explained by the possibility that the mouse testis is better equipped with antiviral machineries than the human testis. This explanation is supported by an early observation that MuV induced weaken antiviral responses in human LCs than mouse counterparts (69). The antiviral abilities of the human and mouse testes are worthy of further comparison, and how the mouse models present human diseases should be considered.

MuV-impaired testicular functions have been investigated using mouse models. Testosterone synthesis and sperm production are two major testicular functions. In accordance with a previous observation that MuV decreased testosterone production by human LCs (70), MuV infection of mouse LCs inhibits the expression of key enzymes necessary for testosterone synthesis and reduces testosterone production (68). Testosterone synthesis is decreased by MuV-induced TNF-α and IL-6 expression because the high levels of both TNF-α and IL-6 inhibit steroidogenesis (71). These results correspond to the reduction of testosterone level in MuV orchitis patients (72), suggesting that the results in mouse models can inform human diseases.

Spermatogenesis disruption and generation of autoantibodies against sperm are common features in adult MuV orchitis patients; the underlying mechanisms, however, are unclear (73). By using mouse models, we found that MuV-induced CXCL10 in SCs promotes germ cell apoptosis (74). Moreover, MuV-induced TNF-α impairs BTB integrity and permeability (75). BTB impairment may result in the release of antigens from the late stages of germ cells behind the BTB to immune components in the interstitial spaces, thereby leading to the production of sperm autoantibodies. The disruption of spermatogenesis after MuV infection should be attributable to the combination effects of the testosterone reduction, germ cell apoptosis, and BTB impairment.

### 3.3 Hepatitis viruses

Hepatitis B virus (HBV) and hepatitis C virus (HCV) are among the most concerning pathogens due to their severe impact on the liver. Both HBV and HCV primarily target the liver and specifically induce hepatitis. HBV has been detected in multiple extra-liver organs including the testis (76, 77), which expresses asialoglycoprotein receptors that mediate HBV infection (78). However, HBV does not impair testicular functions. In contrast, HCV is not detected in the testis. However, both HBV and HCV are detected in the semen and are sexually transmitted. HBV infection may impair sperm quality through the induction of reactive oxygen species (ROS) and the alteration of sperm membrane permeability, thereby disrupting sperm viability, motility, and morphology (79–81). HCV infection is also associated with sperm parameter alteration, including abnormal morphology, decreased sperm number, decreased motility, and sperm death (82, 83). ROS levels and aneuploidy ratios are augmented in the testis of HCV patients (84). Since HCV cases are lower as compared to HBV-infected populations, HBV generally has a higher risk for male infertility than HCV infection.

### 3.4 Human papillomavirus (HPV)

HPV belongs to the papillomaviridae family and infects a large population, with a higher frequency in women than in men (85). HPV infection is a major etiological factor of cervical cancer (86). HPV can be detected in most organs of the MRS and is sexually transmitted (87, 88). HPV infection is generally asymptomatic in men. However, HPV infection is a risk factor of penile squamous cell carcinoma and prostate cancer aggressiveness (89, 90). HPV DNA can be detected in testicular biopsies, mainly in Leydig cells, Sertoli cells, and probably germ cells (91). Convincing evidences demonstrates that HPV impairs semen quality and male fertility (92, 93). HPV infection can disrupt sperm DNA integrity and leading to cells apoptosis (93). Semen infection of HPV significantly impair sperm parameters, including sperm mobility, sperm counts, amplitude of lateral head displacement (94–96). In addition, HPV infection results in the production of autoantibodies against sperm (97).

### 3.5 ZIKA virus (ZIKV)

ZIKV is a mosquito-borne RNA flavivirus that caused an outbreak in South America during 2015–2016, and it has been considered as an international health emergency because it causes severe neurological disorders and neonatal malformations (98). ZIKV persists for a longer duration in the semen and is sexually transmittable, suggesting that the MRS may serve as a viral reservoir (99, 100). Studies using mouse models have demonstrated that ZIKV has a tropism for the testis and impairs testicular functions (101–103). ZIKV infects most mouse testicular cells, including LCs, SCs, MPCs, and germ cells, and induces the expression of inflammatory cytokines in SCs and LCs (102). Most studies on the mechanisms underlying ZIKV infection of testicular cells have focused on SCs. Both mouse and human SCs are susceptible to ZIKV infection (104–106). ZIKV infection of human SCs impairs the BTB integrity *in vitro* (107). The Axl receptor tyrosine kinase promotes ZIKV infection of SCs through the inhibition of cellular antiviral responses (108). Sialic acid facilitates ZIKV internalization into Vero cells (which are derived from the kidney of an African green monkey) and is abundant on the surface of SCs (64, 109). The role of sialic acid in mediating ZIKV infection of SCs remains to be clarified. Moreover, ZIKV infects human and mouse TGCs (110, 111). Intriguingly, human and mouse germ cells support long-term ZIKV replication (111). Since the majority of testicular germ cells reside behind the BTB to escape from immune surveillance, these cells may serve as viral reservoirs. Therefore, ZIKV may be sexually transmitted using germ cells as vectors.

### 3.6 Severe acute respiratory syndrome coronavirus-2 (SARS-CoV-2)

SARS-CoV-2, a new member of single-stranded RNA coronaviruses, emerged at the end of 2019 and rapidly caused the global Coronavirus Disease 2019 (COVID-19) pandemic. While acute respiratory syndrome is the primary manifestation after SARS-CoV-2 infection, many other disorders in almost all systems have been observed. Of them, the adverse effects on reproduction are a major concern (112). In particular, the impacts of SARS-CoV-2 on the male reproductive health have been previously reported (113).

SARS-CoV-2 infection of host cells requires the co-expression of the viral receptors angiotensin-converting enzyme 2 (ACE2) and transmembrane protease serine 2 (TMPRSS 2) (114). The expression of ACE2 was first reported in human testicular LCs (115). A recent study demonstrated that ACE2 is expressed in various testicular cell types, including LCs, SCs, and TGCs (116). By contrast, TMPRSS 2 is faintly expressed, with a very low overlap with ACE2 in human testicular cells (117). Based on the expression pattern of its receptors, SARS-CoV-2 should infect testicular cells. Accordingly, SARS-CoV-2 RNA was detected in only one of 12 testicular tissues from patients that died from COVID-19 (118). However, a very recent report showed that SARS-CoV-2 infected, replicated and activated immune cells in the human testis, suggesting that the testis could be a reservoir for SARS-CoV-2 (119). Remarkably, a robust SARS-CoV-2 signal was observed in the testis and penile of rhesus macaques after infection, supporting that it may be sexually transmitted (120).

The adverse effects of SARS-CoV-2 on the MRS are evident. During the early stages of the pandemic, testicular pain and scrotal discomfort were frequently reported symptoms of SARS-CoV-2 infection in young men (121, 122), suggesting potential inflammatory responses in the MRS (123). Several studies found orchitis and testicular damages in postmortem biopsies of COVID-19 patients (118, 124–127). These observations correspond to the development of orchitis in SARS patients (128). Mechanisms underlying orchitis and testicular damage in COVID-19 and SARS patients remain unclear. Several factors may contribute to SARS-CoV-2-induced orchitis and testicular dysfunction (1): The local infection of virus directly induces inflammation and results in testicular damage (2). Inflammatory cytokine storm in the blood is a major event in the acute cases of COVID-19 and is an important lethal factor. Systemic inflammatory cytokines, such as TNF-α, IL-1, and IL-6, may diffuse to the testis and induce viral pathogenesis because high levels of these cytokines disrupt testicular functions and facilitate inflammatory conditions (129, 130) (3). The fever caused by SARS-CoV-2 infection may impair TGC development since spermatogenesis requires a low temperature as compared to body heat under physiological conditions (4). It has been shown that systemic infection impairs testicular functions (131), and this mechanism may also be involved in the testicular dysfunction in some COVID-19 patients. Notably, the donor age of postmortem biopsies should be considered because testicular function and inflammatory levels can be influenced by age. Moreover, the testicular structure can be damaged by the postmortem duration, as well as the procedures used to collect the biopsies due to its elaborate tissue structure. Therefore, appropriate controls are important in order to draw conclusions.

Several studies have examined the SARS-CoV-2 infection of extra-testicular male reproductive organs, including the epididymis, seminal vesicle, and prostate. Bioinformatics data have shown low levels of ACE2 and TMPRESS2 in these organs (https://www.proteinatlas.org/ENSG00000130234-ACE2/tissue). Accordingly, SARS-CoV-2 was not detected in the prostatic secretion of COVID-19 patients (132, 133), and its pathogenesis in the epididymis, seminal vesicle, and prostate has not been reported. However, various groups have analyzed the potential presence of SARS-CoV-2 in the semen due to the concern of sexual transmission. Most studies support the absence of virus in the semen of COVID-19 patients, except one report that detected SARS-CoV-2 RNA in 6 of 38 semen samples from COVID-19 patients (134). Evidence supporting the sexual transmission of SARS-CoV-2 is missing. Taken together, the detrimental effects of SARS-CoV-2 infection on the MRS remain controversial (135). We can speculate that multiple factors, including symptom severity, treatment drug, and mental stress, in COVID-19 may indirectly impact male reproductive health. Further investigation is required to clarify the effects of SARS-CoV-2 infection on male fertility.

### 3.7 Monkeypox virus (MPXV)

Since May 2022, an outbreak of human MPXV has been rapidly spreading in many countries in Western Europe and the America (136–144). The World Health Organization declared MPXV a public health emergency of international concern on July 23, 2022. Up to the beginning of September 2022, more than 50,000 MPXV cases, including 16 deaths, have been reported in 120 countries. Most notably, MPXV DNA was detected in 90% of semen samples from infected persons, and sexual transmission occurred in 95% cases (145). In Spain and Germany, MPXV was predominantly transmitted among men who had sex with men (130, 131). Similarly, a study from Italy reported four MPXV cases of young adult men who had experienced condomless homosexual intercourse, and MPXV DNA was detected in the semen (136). Some MPXV patients had co-infection with HIV (136, 141). This evidence supports MPXV transmission through sexual contact. Samples obtained from the skin, penile and anal lesions, semen, serum, plasma, feces, and nasopharynx were positive for viral DNA (136). While MPXV may be transmitted through various routes, sexual contact may be a major route. The targets of MPXV in the MRS and its effects on male fertility require urgent investigation.

### 3.8 Other traditional viruses

Herpes simplex virus (HSV)-1 and HSV-2 are among the most common viruses in human beings and are sexually transmitted (146). HSV-1 frequently causes sores in the mouth and occasionally in the genital region, whereas HSV-2 has a tropism for the genital tract and induces herpes in the lower genital tract (147). HSV-2 can be internalized into sperm and detected in the semen (148, 149). HSV-2 infection is associated with abnormal sperm parameters and rarely impairs male fertility (3).

While some other viruses, such as SARS-CoV, Ebola virus (EBOV), and Coxsackie Virus B 5 (CoxVB5), can be detected in the testis, their effects on male fertility are not defined, except for case reports of orchitis in CoxVB5-infected individuals and SARS patients (150–152). The potential detrimental effects of these viruses on male fertility require further investigation. Due to the detrimental impacts of transitional viruses on male fertility and sexual transmission, recent emerging viruses raise the same concerns.

## 4 Innate antiviral defense in the testis

While a large spectrum of viruses have been detected in the testis, only a minority of these viruses replicate and induce pathogenesis. The local innate antiviral responses play roles in the testicular defense against invading viruses. Most testicular cells, including TMs, LCs, SCs, and TGCs, are equipped with innate antiviral machineries.

### 4.1 Antiviral response in TMs

Similar to other macrophages, TMs have phagocytic and bactericidal functions. Although a large number of TMs reside in the interstitial spaces of the testis, these macrophages express relatively low levels of PRRs compared to their counterparts in the circulation and other organs (36). TLR4 levels in rat TMs were significantly lower than that in peritoneal macrophages (38). TMs express low levels of TLR signaling genes and high levels of inhibitory genes of TLR signaling pathways (38). Therefore, the innate immune response induced by microorganisms in TMs is relatively weak as compared with those in their counterparts in other tissues. However, TMs produce anti-inflammatory factors in commitment to the immune privileged status of the testis. TMs and other testicular cells express IFNs and antiviral proteins under physiological conditions and in response to virus challenge, which play roles in the testicular defense against invading viruses (153–155). In general, TMs exhibit an immunosuppressive phenotype to favor a testicular-immune privileged status (36).

### 4.2 Innate antiviral machineries in LCs

The main function of LCs is the production of testosterone. However, increasing evidence shows that LCs also play a central role in regulating the testicular defense against invading viruses (30). The antiviral responses of testicular cells were demonstrated 2 decades ago (156, 157). Rat LCs and other testicular cells constitutively express IFNs and antiviral proteins (153). However, human LCs express relatively low levels of IFNs and antiviral proteins compared with their rat counterparts, suggesting that the innate antiviral response is weaker in the human testis than in the rat testis (69). This observation may explain why certain viruses induce orchitis and impair testicular function in human beings, whereas the virus-induced orchitis and testicular dysfunction are yet to be observed in murine models. Further understanding of the mechanisms underlying the different antiviral response of murine and human LCs can aid in the development of preventive and therapeutic approaches for viral orchitis.

**Figure 3:**
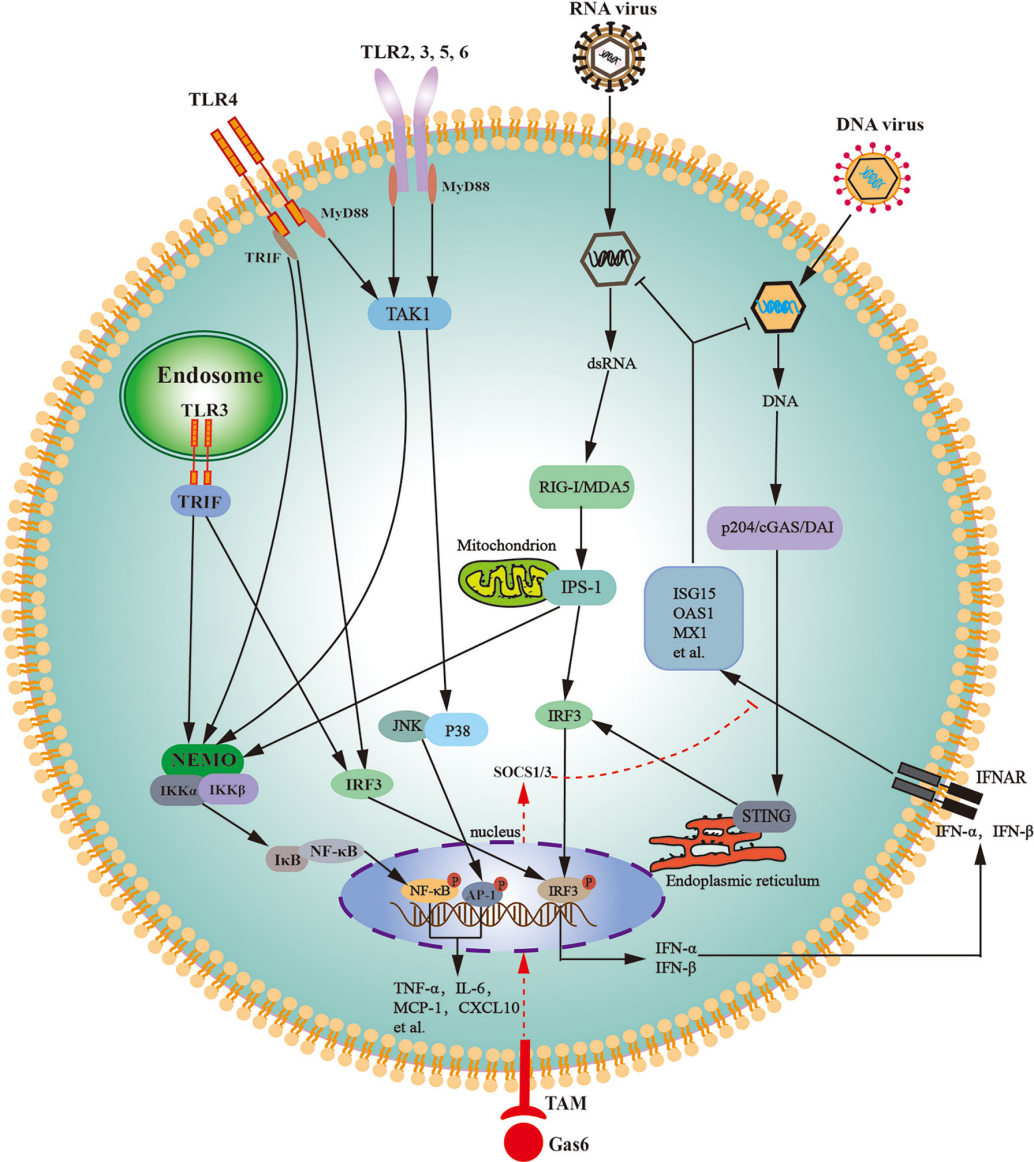
Schematic of the innate immune signaling pathways in Leydig cells. Various toll-like receptors (TLRs), except for TLR3 and TLR4, initiate the MyD88-dependent signaling pathway. TLR3 initiates the TRIF-dependent pathway, whereas TLR4 initiates both MyD88- and TRIF-dependent pathways. The MyD88-dependent pathway induces the expression of proinflammatory cytokines (TNF-α, IL-6) and chemokines (MCP-1, CXCL10) through activation of NF-κB and AP-1. The TRIF-dependent pathway induces the expression of proinflammatory cytokines, chemokines, and type 1 interferons (IFN-α and IFN-β) *via* the activation of NF-κB and IRF3. Cytosolic RNA sensors RIG-I and MDA5 initiate innate antiviral responses through the activation of IPS-1 located on the outer membrane of the mitochondria. The IPS-1-dependent pathway induces the expression of proinflammatory cytokines and chemokines through NF-κB activation and also induces the expression of type 1 interferons through IRF3 activation. DNA sensors p204, cGAS, and DAI trigger antiviral responses through the activation of STING localized in the endoplasmic reticulum. The STING-dependent signaling pathway predominantly induces type 1 interferon expression. Type 1 interferons induce the expression of the antiviral proteins, including ISG15, OAS1, and MX1, in autocrine and paracrine manners. Tyro3, Axl and Mer receptor tyrosine kinases (TAM) can be activated by their common ligand Gas6, which initiates signaling pathway to induce the expression of SOCS1/3, and SOCS1/3 inhibit IFN-triggered antiviral responses. AP-1, activator protein 1; cGAS, cyclic GMP-AMP synthase; CXCL10, C-X-C motif chemokine ligand 10; DAI, DNA-dependent activator of IFN regulatory factors; Gas6, growth-arrest-specific gene 6; IL-6, interleukin-6; IPS-1, IFN-β promoter stimulator 1; IRF3, interferon regulatory factor 3; JNK, Jun amino-terminal kinase; ISG15, IFN-stimulating gene 15; MCP-1, monocyte chemotactic protein 1; MDA5, melanoma differentiation-associated protein 5; MX1, MX GTPase 1; MyD88, myeloid differentiation protein 88; NEMO, NF-κB-essential modulator; IKK, IκB kinase; NF-κB, nuclear factor kappa B; OAS1, 2’5’-oligoadenylate synthetase 1; p204, IFN-inducible protein in mouse; RIG-I, retinoic acid-inducible gene I; SOCS, suppressor of cytokine signaling; STING, stimulator of IFN gene; TRIF, toll/IL-1R domain-containing adaptor inducing IFN-β; TAK1, Transforming-growth-factor-beta-activated kinase; TNF-α, tumor necrosis factor-α.

In agreement with their antiviral responses, LCs abundantly express various PRRs that trigger antiviral signaling. Mouse LCs abundantly express TLR3 and TLR4, which can be activated by their respective ligands and can trigger innate immune responses (158). TLR-initiated innate immune responses in LCs lead to the expression of various proinflammatory cytokines and IFNs through the activation of NF-κB and interferon regulatory factor (IRF3) (**Figure 3**). TAM signaling pathway inhibits TLR3- and TLR4-initiated innate immune responses in LCs (159). A recent study confirmed that Axl and Mer receptors facilitate MuV replication in LCs by inhibiting the cellular innate antiviral response (64).

In addition to TLRs, mouse LCs constitutively express cytosolic viral RNA sensors RIG-I and melanoma differentiation-associated protein 5 (MDA5) (160). Both RIG-I and MDA5 initiate signaling through IFN-β promoter stimulator-1 (IPS-1) after challenge with ligand dsRNA (**Figure 3**). The IPS-1-dependent signaling activates IRF3 and NF-κB, thereby inducing the expression of IFNs and proinflammatory cytokines. IFNs subsequently induce the expression of several antiviral proteins, including 2′5′-oligoadenylate synthetase (OAS1), MxGTPase1 (Mx1), and IFN-stimulating gene 15 (ISG15) in LCs. These antiviral proteins can amplify antiviral signaling, degrade viral RNA, and inhibit viral gene transcription, thereby limiting viral replication in host cells (161). RIG-I- and MDA5-initiated IPS-1 signaling in LCs inhibits testosterone synthesis, indicating that RNA-triggered innate antiviral response impairs testicular functions (160). TLR3-, RIG-I-, and MDA5-initiated innate immune responses would be the mechanism by which RNA viruses, such as MuV, HIV-1, and ZIKV, frequently induce testicular dysfunction.

Cytosolic DNA sensor p204 and its signaling adaptor STING are constitutively expressed in mouse LCs (162). DNA sensor signaling pathways induce type 1 IFNs and antiviral protein expression through activating IRF3 (**Figure 3**). In comparison with RNA, viral DNA induces relative low levels of proinflammatory cytokines and high levels of antiviral proteins (163). This finding corresponds to the fact that various RNA viruses, rather than DNA viruses, frequently induce orchitis and impair testicular functions. Therefore, the manipulation of DNA sensor signaling could be an ideal strategy for enhancing the testicular defense against viral infection and protecting testicular functions. The high level of IFNs produced by LCs may induce the expression of antiviral protein in other testicular cells in a paracrine manner. As such, LCs would play a central role in testicular antiviral defense.

### 4.3 Innate immune response of SCs

Mouse SCs express TLR2–6, and these TLRs initiate innate immune responses to induce the expression of numerous immunoregulatory cytokines (164). These cytokines may promote immune cell recruitment, thereby resulting in testicular inflammation. In particular, TLR3 initiates innate antiviral response in SCs and subsequently induces the production of IFNs and proinflammatory cytokines (165).

In addition to TLRs, various cytosolic DNA and RNA sensors are also expressed in SCs, suggesting that SCs are equipped with innate antiviral machineries. However, the expression levels of cytosolic DNA and RNA sensors are relatively low in SCs compared to LCs (160, 162), indicating that LCs adopt a more efficient antiviral response than SCs. Accordingly, LCs express relatively high levels of IFNs compared to SCs after stimulation by MuV, and MuV replicates more efficiently in SCs than in LCs (57).

### 4.4 Antiviral machineries of TGCs

Although TGCs reside in the seminiferous tubules, viruses from the blood circulation and the ascending genital tract can reach the TGCs in both the basal and adluminal compartments. Early stages of mouse TGCs produce IFNs after stimulation with Sendai virus, and the spermatogonia constitutively express IFN-α, IFN-γ and antiviral proteins, suggesting that TGCs adopt innate antiviral defense (153, 157, 166). TLR3 is expressed during the early stage of mouse TGCs and initiates innate antiviral responses to induce the expression of IFNs and antiviral proteins (167). Since the spermatogonia and spermatocytes are localized both inside and outside of the BTB, TLR3-initiated antiviral response in the TGCs should contribute to the seminiferous epithelial defense against invading viruses from the hematogenous dissemination and the ascending genital tract. Melanoma differentiation-associated protein 5 (MDA5), a cytosolic viral RNA sensor, is expressed in spermatids, suggesting that late-stage TGCs are also equipped with innate antiviral machineries (160). Although TLR3 and MDA5 in mouse TGCs may induce the expression of IFNs and antiviral proteins, the expression levels are relatively low in TGCs compared to that in testicular somatic cells (160, 167). Accordingly, TGCs support long-time propagation of ZIKV, coincident with the decreased expression of antiviral proteins (111). In addition, a recent study demonstrated that ZIKV did not induce an antiviral response in human TGCs, and these cells may serve as a reservoir for ZIKV in humans (110, 168). These studies further confirmed that the antiviral responses of human and mouse TGCs would be different.

In addition to the PRR-innate antiviral response, mouse TGCs can resist viral infection through autophagy (65). TGCs are well equipped with autophagic machineries (169). Autophagy is a conserved lysosome-dependent intracellular process for the degradation of aged organelles and nonfunctional protein aggregates, which are involved in multiple pathophysiological conditions (170). Autophagy is also an intracellular innate defense system against invading microbes, including viruses, bacteria, and protozoa (171, 172). We have recently demonstrated that a specific inhibitor for autophagy significantly increased MuV replication in mouse TGCs (65), suggesting that autophagy plays an antiviral role in mouse TGCs. Since autophagy does not induce the production of proinflammatory cytokines that may induce inflammation and impair testicular functions, it would be an ideal antiviral strategy against viral infection without the induction of inflammation. The antiviral defense of TGCs is particularly important for preventing the transmission of viruses using spermatozoa as vectors and requires further investigation.

## 5 Sexual transmission of viruses

While major testicular cells are equipped with antiviral mechanisms, a large spectrum of viruses may infect the testis and persist for an extended duration. In particular, certain viruses infect TGCs and may transmit using spermatozoa as vectors. Moreover, some viruses may infect other organs of the MRS and shed into the semen, thereby being sexually transmitted.

Most viruses predominantly target primary host cells and possess a major transmission pathway. Some viruses may also be transmitted *via* multiple routes. In addition to blood transmission, sexual transmission is one common transmission pathway for a broad range of viruses, including viruses that primarily target the MRS and other systems.

### 5.1 Virus shedding in human semen

Several viruses that primarily target different organs have been detected to persist for a prolonged period in human semen, which increases the risk for the sexual transmission of these viruses (8, 173). In fact, more than half of the 32 viruses found in the semen are sexually transmitted (**Table 1**). The frequencies of viral shedding into the semen vary greatly for different virus types and are associated with systemic virus titers. During the acute stage of infection with a high viremia, HIV, HBV, HCV, ZIKV, and EBOV can be frequently shed into the semen (6). However, the high shedding frequencies of HIV, HBV, and HCV are observed during the chronic stage with a low viremia (6). HIV shedding frequency is increased by a co-infection with other viruses and inflammatory conditions in the genital tract, which may be due to the enhancement of HIV-positive leukocyte recruitment and facilitation of HIV replication (174). HCV shedding in the semen can be affected by co-infection with HIV (175, 176). Seminal HIV and HCV shedding is generally correlated to viremia. The RNA of ZIKV and EBOV can be detected in the semen of some patients more than 1 year after recovery but is undetectable in blood (177). Age and ejaculation rate are associated with ZIKV shedding (178). Although MuV is detected for a prolonged period in the semen of MuV orchitis-epididymitis patients, evidence regarding MuV sexual transmission is still missing (73).

In addition to systemic viruses, some viruses that have tropism for the genital tract are frequently detected in the semen. Both HPV and HSV have infected a large population throughout the world and persist long after infection. Both genital HPV and HSV are predominantly transmitted *via* sexual contact and are frequently detected in the semen (179–181). As members of the herpes virus family, cytomegalovirus (CMV) and Epstein-Barr virus (EBV) are also shed into the semen and are sexually transmittable. Moreover, seminal CMV or EBV shedding increases HIV shedding prevalence (182, 183). While many other viruses can be detected in the semen and some of them can be occasionally transmitted through sexual contact, these viruses are not typically studied because of their low transmission rate and mild pathogenicity (6).

### 5.2 Origin of viruses in semen

The viruses in semen theoretically originate from two ways: viremia and local infection of the genital tract. Systemic viruses may reach the semen through hematogenous dissemination of viral particles and infected cells from the blood. The coincidence of high virus titers in the blood and semen supports hematogenous origin. In particular, HIV, HBV, and HCV levels in the semen are concomitant with high viremia, and these viruses can be transmitted through blood transfusion and sexual contact. It is reasonable to assume that seminal viruses can be disseminated from the circulating blood. However, the persistent seminal shedding of HIV, ZIKV, and EBOV after recovery of viremia suggests a local origin. In a mouse model, ZIKV infects all major organs of the MRS, including the testis, epididymis, seminal vesicle, and prostate (102). Similarly, a recent study demonstrated that the seminal SIV particles and infected cells of macaques after inoculation with SIV originated from the seminal vesicle, vas deferens, and epididymis (184). Accordingly, over 50% of HIV-infected men have different HIV phenotypes in the blood and semen, suggesting that the seminal viruses may have originated from the MRS (185). The presence of TGCs and sperm infected by ZIKV, HIV, and HBV in the semen of infected men confirm that the testis and/or epididymis can be viral sources (54, 110, 186). The detection of ZIKV RNA and virions in the semen of vasectomized men suggests that the lower parts of the MRS, including the seminal vesicle, prostate, and urethra, can also be viral reservoirs (178, 187). HPV and HSV can be detected in most organs of the MRS, with a high prevalence in penile epithelial and mucosa cells (147, 188). Moreover, HPV and HSV can be internalized into spermatozoa (148, 189). Therefore, HPV and HSV in the semen would predominantly originate from the male genital tract.

### 5.3 Major sexually transmitted viruses

Sexual contact is the primary transmission rout for typical sexually transmitted pathogens, such as *Chlamydia trachomatis*, *Neisseria gonorrhoeae*, *Treponema pallidum*, HIV, genital HSV, and HPV (3). Moreover, a large spectrum of viruses that are primarily transmitted by blood and mosquito vector, such as HBV, HCV, EBOV, and ZIKV, can be also transmitted through sexual contact. There are reports regarding the sexual transmission of dengue virus (DENV) and West Nile Virus (WNV) (190, 191). Additionally, although controversial, the potential sexual transmission of SARS-CoV-2 is a concern (192). Therefore, sexual transmission is a common transmission pathway for a large number of viruses (**Table 1**). Viruses in the semen may be sexually transmitted through the infection of free viral particles and by using infected cells as viral vectors. Currently, virus detection in the semen is largely achieved by detecting the viral genome using PCR. However, viral genomes of viruses that are not sexually transmitted can be detected in the semen, such as certain adenoviruses, bunyaviruses, flaviviruses, paramyxovirues, and retroviruses (173). By contrast, infectious particles of ZIKV and EBOV can be detected in the semen, and both ZIKV and EBOV are sexually transmitted (178, 193). Therefore, the detection of infectious viral particles in semen is a feasible approach to predict the sexual transmission of viruses.

Many sexually transmittable viruses can be transmitted using infected cells as vectors. HIV specifically infects CD4^+^ T cells and mononuclear macrophages and can be sexually transmitted by the infected target cells. However, for most viruses that infect TGCs and spermatozoa, sperm may serve as a “Trojan horse” (194) for sexual transmission. By infecting sperm, viruses not only infect sexual partners *via* parallel transmission but are also vertically transmitted to the offspring. Various sexually transmitted viruses, such as HBV, ZIKV, HPV, HSV, CMV, and HIV, can infect or bind to sperm and potentially transmit to the embryo upon fertilization (6). While sperm-mediated vertical transmission is rarely confirmed in human beings, infection by human sperm with HIV, HBV, and HPV may deliver these viruses to hamster oocytes and embryos after *in vitro* fertilization (189, 195, 196). Notably, HBV infection induces sperm apoptosis and reduces the fertilization of human oocytes *in vitro* (197). Similarly, HPV carried by sperm enter fertilized oocytes and disrupt embryonic development (198). Therefore, HBV, HIV, and HPV may be vertically transmitted to offspring. ZIKV is detected in sperm, and the vertical transmission of ZIKV merits investigation (100). The sexual transmission of viruses *via* parallel and vertical modes is of great concerns with an emerging virus outbreak.

In the context with sexual transmission *via* virus-infected cells, viral infection of testicular germ cells and sperm are particularly concerned. Intriguingly, HIV-1 and HBV can be internalized into testicular germ cells and sperm in the absence of typical viral receptors (186, 199). The mechanisms underlying the uptake of HIV-1 and HBV by germ cells remain to be clarified. The low innate antiviral responses of testicular germ cells and their localization behind the BTB would favor these cells as viral reservoirs. HBV infection of sperm not only vertically transmit viruses, but also affect outcome of assisted reproduction (200). In addition to virus-infected leukocytes and testicular germ cells, ZIKV-positive epithelial cells originated from different sites of the MRS were observed in the semen of ZIKV-infected patients, suggesting that other cell types in the MRS may also serve as vectors for sexual transmission of viruses (201). Viral target cells in the MRS are worthy of further clarification.

### 5.4 Effect of semen on viral infection

While half of the 32 viruses detected in semen are sexually transmitted (**Table 1**), only the minority of them, such as HIV, HPV, HBV, and HSV-2, lead to sexually transmitted diseases. The semen is not only a passive vector but also functions as an inhibitor or a facilitator of viral infection and transmission.

#### 5.4.1 Inhibitory effect of semen on viral infection

The transmission efficiencies are generally low, even for the typical sexually transmitted viruses. For example, while sexual transmission is responsible for most new HIV-1 infections, the sexual transmission efficiency of HIV-1 is less than 0.1% (202). In addition to associated factors, such as virus loads in semen and co-existence of other infections in the male and female genital tracts, it is reasonable to speculate that the semen may impact the efficiency of viral sexual transmission. In fact, the semen can impact HIV-1 infection, acting as both an inhibitor and facilitator (202). Early studies have shown an inhibitory effect of semen on HIV-1 infection, in which seminal cationic polypeptides and exosomes inhibited viral infection through the impairment of interactions between viruses and target cells (203, 204). These observations correspond to the low efficiencies of sexual HIV transmission. Similarly, only a few ZIKV-positive semen samples are infectious (178), and seminal extracellular vesicles inhibit ZIKV infection through the impairment of virus binding to target cells (205, 206). Further, human SP inhibits CMV infection in humans (207). We recently characterized a pan-antiviral factor in the SP that inhibits the infection of various viruses, including MuV, DENV2, HSV-1, and adenovirus 5 (AV5) (208). We defined that the antiviral factor in the semen was derived from the prostate and disrupted virus attachment to target cells by acting on viral particles rather than target cells. We excluded the involvement of seminal exosomes, protein and nuclei acid in the antiviral component. These studies suggest that human semen contains different antiviral factors, which are worthy of further characterization.

#### 5.4.2 Facilitation of semen on viral infection

In contrast to the antiviral activities, some studies have elucidated the facilitatory effect of semen on viral infection. An early study demonstrated that semen-derived amyloid fibrils dramatically enhanced HIV infection (209). Subsequent studies have confirmed that the seminal amyloid fibrils also facilitated infection of CMV, HSV, and EBOV (210–212). In addition to amyloid fibrils, fibronectin also facilitate the HIV infection of T lymphocytes through increased cell attachment, and protected virions from degradation (213, 214). Activation of complement molecule in semen may enhance HIV transmission by facilitating HIV infection of epithelial cells and target cells which express complement receptor (215). In addition, TGF-β is abundantly present in semen and can increase CD169 expression on mature DCs, which involved in the capture and transmission of HIV particles (216). These observations suggest that some seminal components may play opposite roles of inhibiting and enhancing viral infection. Notably, early studies have also demonstrated the facilitatory effect of human SP on CMV and HSV infections (210, 211); however, recent studies have demonstrated that SP inhibits CMV and HSV infections (208, 217). The discrepancies in the results of these studies are yet to be explained. We speculate that different target cells and viral strains were used in different studies, which resulted in discrepant conclusions. Nonetheless, the role of semen in regulating viral infection is an interesting issue as it may provide clues to reduce the sexual transmission of viruses.

## 6 Conclusions

The mammalian MRS is composed of various organs, and it possesses special immunological environments for protecting spermatozoa from adverse immune responses and for defending against microbial infections. Viral infection of the MRS is of great concern due to the potential viral reservoirs and sexual transmission. The immune privileged status and local innate antiviral defense are interesting topics that have been intensively investigated. As a typical immune privileged organ, the testis likely serves as a viral reservoir. While other organs in the MRS, including epididymis, seminal vesicles, and prostate, can also be infected by viruses, the local innate antiviral responses in these organs remain largely unclear and require further investigation. Since seminal viruses can be derived from different organs of the MRS, specific host cells for viral reservoirs in the MRS urgently require clarification, which can aid in the development of strategies for destroying viral sanctuaries. The semen is not only a virus vector for sexual transmission but also impacts viral infection and transmission. The specific seminal factors that inhibit or facilitate viral infection and transmission require further identification and characterization. Overall, the immune privileged status and antiviral defense in the MRS, as well as viral reservoirs and sexual transmission of viruses, leave many unanswered questions.

## Author contributions

DH and FW designed the concept and completed the final editing of the manuscript. All authors contributed to the article and approved the submitted version.

## Funding

This work was supported by grants from the National Natural Science Foundation of China (No. 82071633), CAMS Initiative for Innovative Medicine (No. 2022-I2M-000) and the National Key R&D program of China (No. 2018YF1003900).

## Conflict of interest

The authors declare that the research was conducted in the absence of any commercial or financial relationships that could be construed as a potential conflict of interest.

## Publisher’s note

All claims expressed in this article are solely those of the authors and do not necessarily represent those of their affiliated organizations, or those of the publisher, the editors and the reviewers. Any product that may be evaluated in this article, or claim that may be made by its manufacturer, is not guaranteed or endorsed by the publisher.
